# Electromyography, Stiffness and Kinematics of Resisted Sprint Training in the Specialized SKILLRUN^®^ Treadmill Using Different Load Conditions in Rugby Players

**DOI:** 10.3390/s21227482

**Published:** 2021-11-10

**Authors:** Antonio Martínez-Serrano, Elena Marín-Cascales, Konstantinos Spyrou, Tomás T. Freitas, Pedro E. Alcaraz

**Affiliations:** 1 UCAM Research Center for High Performance Sport, Catholic University of Murcia, 30107 Murcia, Spain; amartinez30@ucam.edu (A.M.-S.); kspyrou@ucam.edu (K.S.); palcaraz@ucam.edu (P.E.A.); 2 Strength and Conditioning Society, 00118 Rome, Italy; elenamcascales@gmail.com; 3 Faculty of Sports Sciences, Catholic University of Murcia, 30107 Murcia, Spain; 4 NAR—Nucleus of High Performance in Sport, São Paulo 04753-060, Brazil

**Keywords:** team-sports, performance, muscle activation, loaded sprint, sled-push

## Abstract

This study’s aim was to analyze muscle activation and kinematics of sled-pushing and resisted-parachute sprinting with three load conditions on an instrumentalized SKILLRUN^®^ treadmill. Nine male amateur rugby union players (21.3 ± 4.3 years, 75.8 ± 10.2 kg, 176.6 ± 8.8 cm) performed a sled-push session consisting of three 15-m repetitions at 20%, 55% and 90% body mas and another resisted-parachute session using three different parachute sizes (XS, XL and 3XL). Sprinting kinematics and muscle activity of three lower-limb muscles (biceps femoris (BF), vastus lateralis (VL) and gastrocnemius medialis (GM)) were measured. A repeated-measures analysis of variance (RM-ANOVA) showed that higher loads during the sled-push increased (VL) (*p* ≤ 0.001) and (GM) (*p* ≤ 0.001) but not (BF) (*p* = 0.278) activity. Furthermore, it caused significant changes in sprinting kinematics, stiffness and joint angles. Resisted-parachute sprinting did not change kinematics or muscle activation, despite producing a significant overload (i.e., speed loss). In conclusion, increased sled-push loading caused disruptions in sprinting technique and altered lower-limb muscle activation patterns as opposed to the resisted-parachute. These findings might help practitioners determine the more adequate resisted sprint exercise and load according to the training objective (e.g., power production or speed performance).

## 1. Introduction

Rugby union is a high contact team sport played worldwide which performance depends on the complex relationship between technique, tactics, cognition and physical capacities [1]. The game is based on collision and intermittent actions, where high-intensity activities (e.g., tackling, rucking, scrummaging, mauling) are interspersed with low-intensity activities (e.g., standing, walking, jogging) [2]. By analyzing the activity profile during a rugby union match, high-intensity actions, such as sprinting, are very frequent [3]. As such, linear sprint could be considered one of the most critical skills in this sport [4].

Sprint performance is determined by the athlete’s capacity to generate and apply a great propulsive force during the acceleration phase and to maintain their maximum velocity for as long as possible during the maximum velocity phase [5]. In this regard, different non-specific strength-power exercises and methods have been used for the improvement of the acceleration phase of the sprint [6,7,8]. However, many coaches believe that training methods for improving sprint performance should also include specific strength exercises, so that the athlete can perform the desired movement with an added load [9]. This idea is supported by the training principle of specificity, which suggests that exercises should have similar characteristics to the sport’s requirements (i.e., type of action, movement patterns, velocity, muscle activation, etc.) [10]. Thus, resisted sprint training (RST) has been used as a specific training method for the enhancement of sprint performance in rugby and other team-sports, especially in the acceleration phase [11,12,13,14].

One of the most important variables considering RST is the selection of the training load. Most authors agree that RST is an effective training method for performance improvement, regardless of the load used [6,11,15,16]. Nevertheless, some argue that the use of tertiary methods does not replicate the sprint running movement [14,15] and the load must not be >20% body mass (BM) if the aim is to replicate sprint demands in terms of movement pattern, load, muscle activation and movement velocity [11]. These kinematics changes are mainly caused by a decrease in the lower limb stiffness, leading to a reduction of the force transmission ratio between the legs and the ground and therefore a lower acceleration and running speed [17].

When referring to RST, a wide variety of exercises and equipment can be used including sled and parachute towing, wearing a weighted vest and sprinting on sand or uphill [15]. From these, sled towing and pushing, along with resisted-parachute sprinting, are the most widely used in sports such as football, rugby and soccer. However, the scientific evidence regarding sled-pushing and resisted-parachute sprinting is limited in comparison to sled towing [18,19,20,21,22], particularly for variables such as muscle activation. In fact, only one study has analyzed muscle activation patterns in sled-pushing compared to squatting, finding a similar rectus and biceps femoris (BF) activation but higher gastrocnemius electromyographic (EMG) activity in the sled exercise [20].

A potential limitation of the RST is that it requires an exterior environment and facilities for its development, otherwise, a large interior space is needed. In addition, weather conditions can have a negative effect conducting the workout (e.g., wind conditions). Hence, alternative methods/equipment that can replicate the demands of RST indoor could be extremely valuable for coaches and athletes. In this context, a specialized treadmill SKILLRUN^®^ (SR^®^) (Technogym, Cesena, Italy) capable of replicating RST has been recently developed with the aim of improving athlete’s speed and power in a closed environment.

Given the lack of research, performing a muscle activity and kinematics analysis in sled-pushing and resisted-parachute sprinting on a treadmill with different loads would be interesting to determine which load in each of these exercises allows performing a sprinting effort without major disruptions of the muscle activity, movement pattern and leg stiffness. Hence, the aim of this study was to analyze the muscular activation and kinematics of sled-pushing and resisted-parachute sprinting with three load conditions on the instrumentalized treadmill. The secondary objective was to examine the effect of varying load on power production in these specific exercises. We hypothesized that: (1) the increased load would disrupt the kinematics of the exercises and cause increased gastrocnemius medialis (GM) and vastus lateralis (VL) muscle activation whereas BF would be reduced or maintained; and (2) moderate intensity loads would maximize power production.

## 2. Materials and Methods

**Figure 1:**
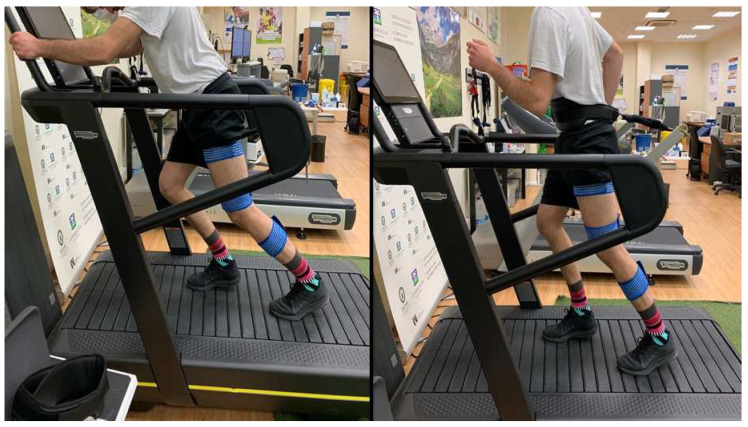
(**left panel**) Sled-push and, (**right panel**) resisted-parachute sprinting on the SR^®^ treadmill.

Participants took part in a randomized crossover design pilot study consisting of: (1) one sled-push session in SR^®^ treadmill using three different load conditions (i.e., 20%, 55% and 90% BM), and (2) one resisted-parachute session in SR^®^ treadmill using three different parachute sizes (i.e., extra-small (XS), extra-large (XL) and triple extra-large (3XL)). Sled and parachute resistance were applied by the SR^®^; therefore, participants did not move across space but rather ran on the treadmill as depicted in Figure 1. Test distance and load selection were determined following a pilot study conducted at our facilities. An external researcher randomly determined the order of the sessions, the training intensity and parachute size. Each sled and parachute session were separated by 7-d due to team’s training schedule during the season. Participants were asked to cease physical activity or training 24-h before the testing to ensure full recovery and all the tests were conducted in a similar time of the day (e.g., +/− 1-h) to minimize diurnal variations.

### 2.1. Subjects

Nine male amateur rugby union players (age 21.3 ± 4.3 years, mass 75.8 ± 10.2 kg, height 176.6 ± 8.8 cm) participated in this study. Convenience sampling was used as the eligibility criteria. Over the course of the study, two participants suffered an injury and were unable to attend to the resisted-parachute session. Players were excluded if they: (1) were taking any medication or supplementation (e.g., caffeine 12-h prior to exercise) that could interfere with the results, (2) were suffering from any kind of disease and (3) had suffered from a lower limb injury six months prior to study enrollment. All subjects were familiar with performing the traditional sled-push and resisted-parachute sprinting exercises in their regular training. Participants read the information sheet and were informed of the benefits and risks of the investigation and signed the informed consent form before the study began. Parental or guardian informed consent form was obtained for those who were underage (*n* = 2). This study conforms with The Code of Ethics of the World Medical Association (Declaration of Helsinki) and it was approved by the local Ethics Committee (code: CE012009; date 31 January 2020).

### 2.2. Procedures

Anthropometric measurements (i.e., mass and height) were taken using a Tanita HD-313 scale (Tanita Corporation, Tokyo, Japan) and a stadiometer Seca 213 (Hamburg, Germany). Electrodes for the EMG analysis were placed on the VL, BF and GM muscles before volunteers performed a standardized warm-up which included: 8-min of cycling in a cycle ergometer, dynamic stretching of the lower limbs and one submaximal sled-push repetition with the participant’s 20% BM over 15-m or a submaximal resisted-parachute sprint using XS parachute size over 15-m.

#### 2.2.1. Sled-Push Test Protocol

In the SR^®^ sled mode, the resistance is applied in such a way that it mimics the sensation of an over-ground sled-push. The treadmill resistance is higher during the initial phase of the run and decreases at a constant rate as velocity increases (accounting for inertia). Participants (*n* = 9) carried out three repetitions over 15-m and used three different training intensities: 20%, 55% and 90% BM. They had to run, “pushing” the treadmill belt, as fast as possible (speed was not kept constant by the treadmill but was rather determined by the athlete’s running capabilities) with their hands fixed to the handles at the height they were most comfortable following manufacturer’s recommendations. Starting position was established individually according to the subject’s dominant leg and remained the same throughout all the sessions. Participants were encouraged to exert their maximum effort while performing the exercises. Resting time between repetitions was 3-min walking at 3 km/h.

#### 2.2.2. Resisted-Parachute Test Protocol

In the SR^®^ parachute mode, the sensation of sprinting outdoors with a parachute is also mimicked. The resistance is null at the start and increases progressively with running velocity. According to manufacturer specifications, the resistance deriving from the parachute is calculated analyzing different parameters (Equations (1)–(3)) that are used into a proprietary formula. The parameters are:

(1) $$\mathrm{Motor}\;\mathrm{torque}=0.01365\times \left(v^{2}\right)\times \left({\mathrm{Pd}}^{2}\right)\times \left(10^{-6}\right)$$

(2) $$\mathrm{Force}\;(N)=\mathrm{FO}+0.615752\times \left({\mathrm{Pd}}^{2}\right)\times \left(v^{2}\right)\times \left(10^{-6}\right)$$

(3) $$\mathrm{Power}\;(W)=\mathrm{PO}+0.615752\times \left({\mathrm{Pd}}^{2}\right)\times \left(v^{3}\right)\times \left(10^{-6}\right)$$

In which F0 corresponds to the friction coefficient in N, v is the slat belt speed in m/s, P0 (W) is obtained by multiplying F0 by v, and Pd corresponds to the parachute diameter in mm. Resistance increases with the power of three relationship with speed (cubic relation).

Participants (*n* = 7) performed three repetitions over 15-m and used three different parachute sizes: XS, XL and 3XL. The parachute belt was buckled at waist level following manufacturer’s recommendations. Participants were asked to run at maximum intensity and were encouraged over the course of the test. Resting time between repetitions was 3-min walking at 3 km/h.

#### 2.2.3. Electromyography

The Surface ElectroMyoGraphy for the Non-Invasive Assessment of Muscles (SENIAM) protocol was used for skin preparation and sensor location [23]. Skin preparation included shaving areas where electrodes would be placed, removing dead epithelial cells using an abrasive paper and cleansing the area with alcohol, allowing it to vaporize. Two surface EMG electrodes (Ambu^®^ BlueSensor N—Ambu A/S, Denmark) were placed 20 mm apart (electrode to electrode) on the participant’s dominant leg over three muscles: (a) VL, (b) BF and (c) GM. The electrodes were placed superficially to each muscle belly and in the same orientation as the respective muscle fibers. This procedure was conducted before the beginning of the sled-push and resisted-parachute session. The placement of the electrodes was marked with a permanent marker to ensure that it was the same in both sessions. They were secured to the skin with adhesive tape and an elastic bandage in order to eliminate any movement artifact.

Muscle activation was measured via wireless surface EMG (Noraxon USA INC, Scottsdale, AZ, USA) at a sampling rate of 10,000 Hz with Noraxon MR 3.6.20 software (Noraxon, Scottsdale, AZ, USA). Raw EMG data was processed and filtered using the following settings: Filter: FIR, Window: 79 points, Type: Bandpass, Low frequency: 20 Hz, High frequency: 500 Hz, Window: Lancosh. Rectification and smoothing (Algorithm: RMS, Window: 100 ms) were also applied. Total muscle activation was analyzed with AcqKnowledge 3.9.1 software (BIOPAC Systems Inc., CA, USA) by calculating the average root-mean-square (RMS) of the whole gait cycle from the first 10 strides.

#### 2.2.4. Performance Variables

Maximum velocity (V_max_) and maximum power (P_max_) were obtained from the specialized treadmill interface as performance variables. According to manufacturer specifications V_max_ (km/h) is directly measured from the rotational speed of the motor while P_max_ (W) is obtained by multiplying the rotational speed of the slat belt by the force applied by the athlete to the surface (deriving from the motor energetic absorption).

#### 2.2.5. Kinematics

Running kinematics during the sled-push and parachute sessions were recorded using the camera of an iPhone XR running iOS 13.5 (Apple Inc., Cupertino, CA, USA) at a frequency of 240 Hz. The camera was placed sideways at a distance of 2-m from the treadmill on a 1-m height tripod recording the sagittal plane of the subject’s dominant leg. Calibration frame was performed by measuring the length of one of the treadmill handles.

The following kinematic variables of the first ten strides of the participant’s dominant leg were analyzed using Kinovea 0.9.1 (Kinovea.org, France): contact time (CT), flight time (FT), stride frequency (SF), stride length (SL), leg stiffness (K_vert_) and ankle, knee and hip angles (A_angle_, K_angle_, H_angle_, respectively) collected during the stance phase. Intraclass correlation coefficients (ICC) were determined for the different sled-push variables: CT (ICC ranging from 0.890 to 0.965), FT (from 0.744 to 0.940) and SL (from 0.883 to 0.945). Regarding resisted-parachute sprinting, ICCs ranging from 0.816 to 0.967, from 0.704 to 0.831 and from 0.765 to 0.911 were obtained for CT, FT and SL, respectively. K_vert_ was measured using the methods and calculations (Equations (4)–(6)) by Morin et al. [24]:

(4) $${\hat{K}}_{\mathrm{vert}}={\hat{F}}_{\max}\cdot \;\Delta {\hat{y}}_{c}{}^{-1}$$

(5) $${\hat{F}}_{\max}=mg\;\frac{\pi }{2}\;\left(\frac{t_{f}}{t_{c}}+1\right)$$

(6) $$\Delta {\hat{y}}_{c}=\frac{{\hat{F}}_{\max}t_{c}^{2}\;}{m\;\pi^{2}}+g\;\frac{t_{c}^{2}}{8}$$

In which $\Delta {\hat{y}}_{c}$ is the vertical center of mass displacement, m is the participant’s body mass in kg, *t_f_* is the flight time in s, and $t_{c}$ is the contact time in s. Subsequently, the ${\hat{K}}_{\mathrm{vert}}$ value obtained was multiplied by 1.0496 (i.e., a correction factor proposed by Coleman et al. [25]). Raw angle data from Kinovea was exported to Microsoft Excel 16.36 (Microsoft, USA) for further analysis.

### 2.3. Statistical Analysis

Data is shown as mean ± SD. The statistical analysis was performed using Jamovi^®^ 1.1.9.0 for macOS. Shapiro–Wilk test and Levene’s test were used for assessing the normality of the distribution of the variables and the homogeneity of variance. The EMG activity and kinematic variables during each load and exercises were determined using repeated-measures analysis of variance with Bonferroni post hoc comparisons. Partial eta squared was obtained from the repeated measures analysis and classified as: small (≤0.01), moderate (≤0.06) and large (≥0.14). Cohen’s d effect sizes (ES) were calculated to provide qualitive descriptors of standardized effects using the following criteria: <0.2, 0.2–0.6, 0.6–1.2, 1.2–2, 2–4 and >4 for trivial, small, moderate, large, very large and near perfect, respectively [26]. Alpha-level was set at *p* ≤ 0.05.

## 3. Results

### 3.1. Electromyography

**Figure 2:**
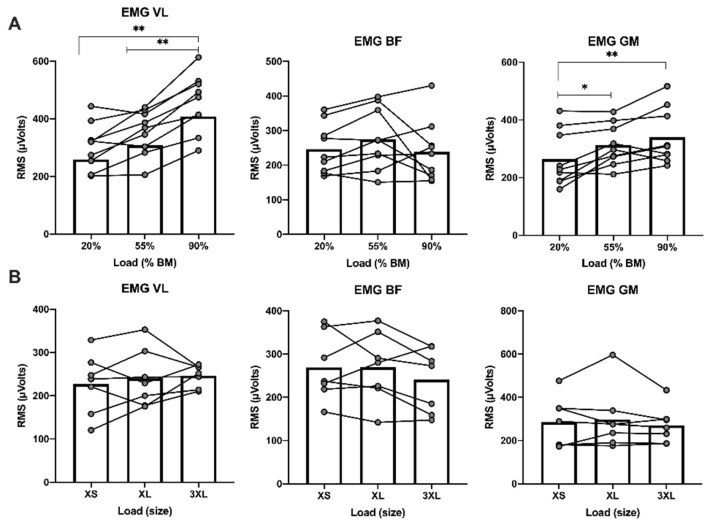
(**A**) Comparison of muscle activation of VL, BF and GM in sled-push under different load conditions. (**B**) Comparison of muscle activation of VL, BF and GM in resisted-parachute sprinting under different size conditions. * *p* ≤ 0.05; ** *p* ≤ 0.001; BF = biceps femoris; BM = body mass; EMG = electromyography; GM = gastrocnemius medialis; VL = vastus.

Figure 2 displays the comparisons between EMG activation patterns of the different muscles in the sled-push and resisted-parachute sprinting in the different load conditions. Regarding sled-push, there was a statistically significant effect of increasing load on VL activation (F = 33.366; *p* ≤ 0.001; η^2^_P_ = 0.807).VL activation was significantly higher at 90% BM compared to 20 and 55% BM (*p* ≤ 0.001, ES = 2.18; *p* ≤ 0.001, ES = 2.39) respectively, and tended to increase from 20–55% BM (*p* = 0.054, ES = 0.90). In contrast, no significant differences were obtained on BF activation as load increased (F = 1.388; *p* = 0.278; η^2^_P_ = 0.148). Increasing load had a statistically significant effect on GM activation (F = 14.439; *p* ≤ 0.001; η^2^_P_ = 0.643). GM activation increased significantly from 20–55% BM (*p* = 0.012, ES = 1.07) and 20–90% BM (*p* ≤ 0.001, ES = 1.94) but not from 55–90% BM (*p* = 0.212, ES = 0.62) (Figure 2A). No significant differences were found in muscle activation of VL (F = 0.591; *p* = 0.569; η^2^_P_ = 0.090), BF (F = 1.531; *p* = 0.256; η^2^_P_ = 0.203) and GM (F = 0.879; *p* = 0.440; 0.128) using different parachute sizes (Figure 2B).

### 3.2. Kinematics

Table 1 depicts the descriptive analysis for the kinematic variables.

**Figure 3:**
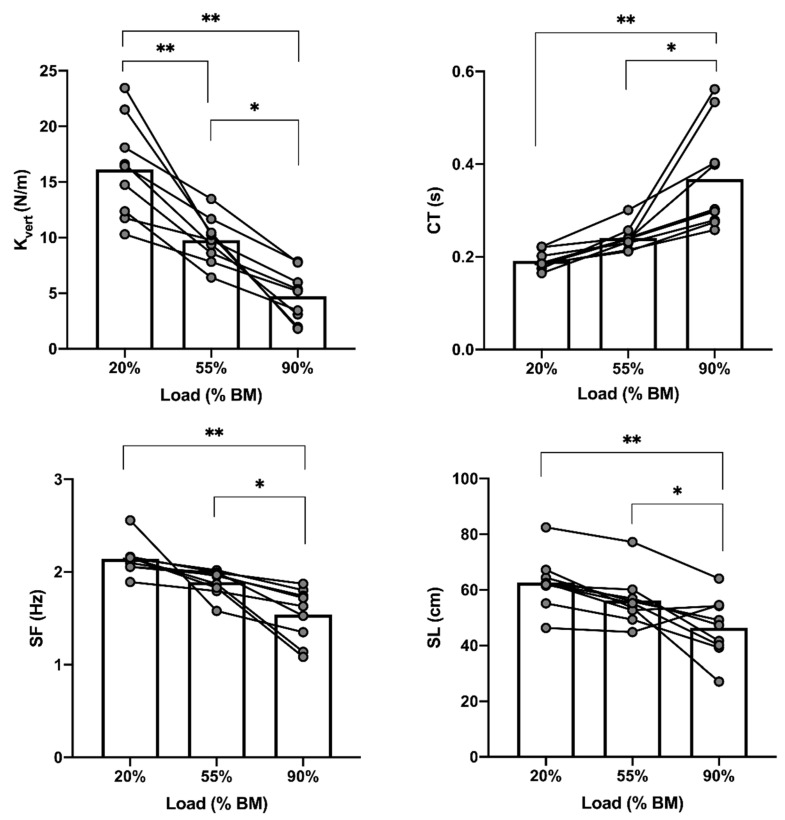
Comparison of sprinting K_vert_ and kinematics under different load conditions in sled-push. * *p* ≤ 0.05; ** *p* ≤ 0.001; BM = body mass; CT = contact time; FT = flight time; K_vert_ = vertical stiffness; SF = stride frequency; SL = stride length.

Significant effects were found in CT (F = 16.367; *p* ≤ 0.001; η^2^_P_ = 0.672), SF (F = 16.543; *p* ≤ 0.001; η^2^_P_ = 0.674), SL (F = 12.505; *p* ≤ 0.001; η^2^_P_ = 0.610) and K_vert_ (F = 33.841; *p* ≤ 0.001; η^2^_P_ = 0.809) when pushing the sled. Higher CT were found from 20–90% BM (*p* ≤ 0.001, ES = 1.42) and 55–90% BM (*p* = 0.003, ES = 1.20). Conversely, no changes were found in FT (F = 1.130; *p* = 0.347; η^2^_P_ = 0.124). SF and SL increased significantly from 20–90 % BM (*p* ≤ 0.001, ES = 1.52; *p* ≤ 0.001, ES = 1.28) and 55–90% BM (*p* = 0.013, ES = 1.44; *p* = 0.025, ES = 0.92), respectively. K_vert_ decreased significantly in all load conditions 20–55% BM (*p* ≤ 0.001, ES = 1.72), 20–90% BM (*p* ≤ 0.001, ES = 1.98) and 55–90% BM (*p* = 0.007, ES = 2.21) (Figure 3).

Increasing load had a significant effect on A_angle_ (F = 12.075; *p* ≤ 0.001; η^2^_P_ = 0.601), K_angle_ (F = 10.088; *p* = 0.001; η^2^_P_ = 0.558) and H_angle_ (F = 4.611; *p* = 0.026; η^2^_P_ = 0.366). A_angle_, K_angle_ and H_angle_ decreased significantly from 20–90% BM (*p* ≤ 0.001, ES = 1.46; *p* = 0.001, ES = 1.14; *p* = 0.031, ES = 0.79, respectively) and presented a non-significant decrease from 55–90% BM in A_angle_ (*p* = 0.062, ES = 0.99). No significant effects between kinematic variables and different parachute sizes in resisted-parachute sprinting were found in CT (F = 2.982; *p* = 0.089; η^2^_P_ = 0.332), FT (F = 0.541; *p* = 0.595; η^2^_P_ = 0.083), SF (F = 0.416; *p* = 0.669; 0.065), SL (F = 3.568; *p* = 0.061; 0.373) and K_vert_ (F = 3.109; *p* = 0.082; η^2^_P_ = 0.341). The only statistically significant difference was found from XL-3XL parachute size in H_angle_ (*p* = 0.007, ES = 1.64).

### 3.3. Performance

In sled-push, there were a statistically significant effect of increasing load on P_max_ (F = 27.101; *p* ≤ 0.001; η^2^_P_ = 0.772) and V_max_ (F = 86.972; *p* ≤ 0.001; η^2^_P_ = 0.916). P_max_ increased significantly from 20–55 % BM (*p* ≤ 0.001, ES = 4.80), 20–90 % BM (*p* = 0.001, ES = 1.26) and decreased significantly from 55–90% BM (*p* = 0.040, ES = 0.81). On the other hand, V_max_ declined significantly between 20–55% BM (*p* ≤ 0.001; ES = 3.35), 20–90% BM (*p* ≤ 0.001; ES = 3.78) and 55–90% BM (*p* ≤ 0.001; ES = 2.02). During resisted-parachute sprinting, we found a significant effect on P_max_ (F = 30.934; *p* ≤ 0.001; η^2^_P_ = 0.838) and V_max_ (F = 20.541; *p* ≤ 0.001; η^2^_P_ = 0.774). P_max_ increased significantly from XS to 3XL (*p* ≤ 0.001, ES = 2.75) and XL to 3XL (*p* ≤ 0.001, ES = 2.68), whereas V_max_ decreased significantly between XS-XL (*p* = 0.003, ES = 1.99) and XS-3XL (*p* ≤ 0.001, ES = 1.91).

## 4. Discussion

The main findings of the study were that: (1) the muscle activation of the VL and GM (but not the BF) increased as a function of the load while pushing the sled but not when using parachutes of different sizes; (2) increasing the load in sled-push provoked several changes in running kinematics (i.e., increased CT and decreased SF, SL and K_vert_, A_angle_, K_angle_ and H_angle_) whereas only an increase in the H_angle_ between XL-3XL sizes was detected in parachute running; and (3) the load conditions that produced the highest power output in sled-push and parachute were 55% BM and 3XL parachute size, respectively.

The reported EMG activity in VL while pushing the sled is supported by previous evidence suggesting there is an increase in knee torque due to increased horizontal concentric force during the acceleration phase of the sprint [27,28]. During this phase, the position of the trunk is leaning forward, bringing the body to a more horizontal position [29], similar to the one adopted to push the sled. In RST, athletes must adopt a more horizontal position [30] and lower their center of mass to increase the horizontal force application and ground CT and overcome the load [31]. This movement pattern defined as “Groucho running” [32] (i.e., increased trunk, knee and ankle flexion while running) could explain why VL and GM activation increased in all load condition whereas BF remained unchanged. Regarding GM, it is worth noting that this muscle plays an important role in the vertical and horizontal acceleration profiles during the stance phase in sprint acceleration [33]. The increased GM activity with heavier loads could be explained by its function as a dynamic muscle and by being the last segment of the kinetic chain trying to maintain linear momentum [20]. The present data is supported, at least in part, by Zabaloy et al. [34] that analyzed and compared the effects of unresisted and RST with 0%, 10%, 30% and 50% velocity loss (V_loss_) in rugby players. The authors found that BF long head EMG decreased significantly as sled load increased whereas RF EMG increased. However, they did not notice any significant changes in GM and gluteus medius. Regarding resisted-parachute sprinting, it could be interesting to observe that EMG activity of the analyzed muscles remained unchanged during with different sizes. This might be related to the fact that no significant changes in running kinematics were found, despite the observed decrease in V_max_. Still, these findings should be taken with caution as, to our knowledge, this is the first study investigating muscle activation in parachute-resisted sprinting.

Regarding kinematic analysis, the increased load caused a disruption in most variables during the sled-push. CT increased in all load conditions, as the athlete was “forced” to produce a greater muscular power and horizontal force at ground contact to overcome the higher resistance [30,34]. SL decreased even though no change in FT was found. This is not related to the idea that shorter SL is associated with decreased FT [30]. However, this exercise was performed on a treadmill; therefore, the relationship between the kinematic variables could be different than if it had been carried out overground [35]. Concerning the parachute condition, the findings herein are consistent with previous research [21], that reported that, despite parachute sprinting speed significantly decreasing by 4.4%, SF, SL, ground CT and joint angles (trunk, hip, knee and ankle) remained unchanged. In line with these results, Alcaraz et al. [15] established a 5% decreased running velocity in men and 6% on women with a medium size parachute compared to an unload sprint. Therefore, it appears that resisted-parachute sprinting caused an overload on the athlete without changing running kinematics and muscle activation patterns.

K_leg_ is a variable that plays an important role in sprint performance as it is associated with velocity, SF and energy cost [24]. In this regard, in the present study, K_vert_ decreased significantly with increasing loads. Nevertheless, caution is necessary when comparing sled-pushing and sled pulling since, despite both being effective RST exercises, they may offer different training stimuli [18]. Another aspect worth noting is that the significant reduction in A_angle_, K_angle_ and H_angle_ herein could lead to an increased energy cost of the movement pattern as a result of a decline in the amount of stored and reused elastic energy [36]. This, together with an alteration of running kinematics and greater moments of force caused by the increased load, could raise the risk of sustaining an injury [37].

Of note, no previous research explored the use of different loads in sled-push and parachute running. Different authors have addressed this issue in other sled-resisted exercises (e.g., sled towing). For example, Cross et al. [38], using a sled towing protocol, found a range from 70–96% BM (recreational athletes: 70%; sprinters: 96%) to be optimal for power production. Opposite to these findings, Monte et al. [39] established maximal horizontal power production in male sprint athletes at 20% BM. In this study, although all kinematic parameters changed significantly with external load (CT, FT and SL), there was no variation in the angular parameters (i.e., in running technique). Importantly, caution is needed when discussing these values as optimal load is considered to be exercise-specific, therefore, the same relative intensity should not be applied to all sled-resisted exercises [40]. This could be explained by the fact that power production is affected by the biomechanical and neurophysiological characteristics of each exercise and the intrinsic characteristics of the athlete himself (training background, hypertrophy, distribution and type of fibers) [40,41]. Determining the load that maximized power production can be beneficial for programming the training; however, it is yet to be determined whether training with the optimal load in RST yields greater adaptations.

The main limitation of the present study is the small and heterogeneous sample size. A larger sample would have allowed us to get more statistical power. In addition, the non-normalization of muscle activation values could be considered a limitation. Nevertheless, the experimental context herein (i.e., comparison within a person and muscle, between different loads (within a session) without removing electrodes) allows the approach used (non-normalized data), as discussed elsewhere [42]. Future research should analyze the pattern of muscle activation during the different phases of the gait cycle while pushing the sled and sprinting with parachute so that it is possible to understand in which phases the lower limb muscles are more involved. Moreover, it would be interesting to study the long-term effects of RST on a variety of sport modalities (e.g., team-sports, athletics or endurance athletes).

## 5. Conclusions

In conclusion, the increased load in sled-push causes a disturbance in sprinting technique accompanied by changes in lower-limb muscle activation patterns. Conversely, sprinting with different parachute sizes does not change running kinematics and muscle activation, but it causes and overload on the athlete by increasing V_loss_. As hypothesized, the load that maximized power production in sled-pushing was found at 55% BM. In resisted-parachute sprinting the biggest parachute size produced the highest power output.

From a practical perspective and based on our findings, increased load during the sled-push exercise in SR^®^ treadmill modifies muscle activation, stiffness and kinematics. Therefore, depending on the training objective, we recommend strength and conditioning professionals to use: (1) very high loads (i.e., around 90% BM) to maximize the activation of the quadriceps and gastrocnemius muscles, (2) loads around 55% BM to maximize power production and (3) loads below or close to 20% BM if the objective is to improve velocity. Moreover, resisted-parachute sprinting in the SR^®^ treadmill could be useful for improving sprint force production without compromising sprinting kinematics. The SR^®^ treadmill was found to acutely modify muscle activation patterns and force production against the ground when performing RST. Therefore, this specialized treadmill seems to be a highly versatile device for training in different zones of the force-velocity curve.

## Figures and Tables

**Table 1:** Kinematics and performance variables of sled push and resisted-parachute sprinting with different load conditions, data is presented as mean ± SD.

	Sled Push			Parachute		
	20% BM	55% BM	90% BM	XS	XL	3XL
**Kinematic Variables**						
**CT (s)**	0.192 ± 0.012	0.241 ± 0.026	0.368 ± 0.115 **	0.186 ± 0.012	0.197 ± 0.009	0.196 ± 0.016
**FT (s)**	0.297 ± 0.019	0.291 ± 0.025	0.305 ± 0.042	0.283 ± 0.018	0.277 ± 0.016	0.279 ± 0.026
**SF (Hz)**	2.14 ± 0.18	1.89 ± 0.14	1.54 ± 0.29 **	2.13 ± 0.09	2.11 ± 0.08	2.11 ± 0.10
**SL (cm)**	62.63 ± 9.64	56.21 ± 9.05	46.39 ± 10.8 **	59.63 ± 7.41	58.43 ± 6.36	54.25 ± 5.49
**K_vert_ (N/m)**	16.14 ± 4.42	9.76 ± 2.08 **	4.72 ± 2.28 **	16.48 ± 4.33	14.37 ± 3.19	14.83 ± 4.02
**Joint Angles**						
**A_angle_ (º)**	106.73 ± 7.88	103.05 ± 11.04	99 ± 8.95 **	110.60 ± 2.99	108.76 ± 5.92	112.25 ± 6.58
**K_angle_ (º)**	142.27 ± 8.21	135.52 ± 9.64	127.63 ± 13.03 *	143.46 ± 11.07	141.30 ± 12.44	148.87 ± 7.03
**H_angle_ (º)**	142.52 ± 6.11	140.73 ± 10.69	135.03 ± 12.29 *	151.99 ± 6.50	149.37 ± 4.72	157.09 ± 3.78 *
**Performance Variables**						
**P_max_ (W)**	704.56 ± 107.37	900.89 ± 132.89 **	826.00 ± 121.04 *	440.71 ± 93.08	469.71 ± 85.19	533.14 ± 80.83 **
**V_max_ (km/h)**	17.36 ± 1.03	13.19 ± 1.02 **	8.81 ± 2.62 **	18.83 ± 1.62	16.80 ± 1.69*	15.96 ± 1.36 **

* *p* ≤ 0.05; ** *p* ≤ 0.001; η^2^_P_ = significant difference between XL-3XL Aangle = ankle angle; BM = body mass; cm = centimeters; CT = contact time; FT = flight time; Hangle = hip angle; Hz = hertz; km/h = kilometers per hour; Kangle = knee angle; Kvert = stiffness vertical; s = seconds; SF = stride frequency; SL = stride length; Vmax = maximum velocity; W = watts; XS = extra-small; XL = extra-large; 3XL = triple extra-large.

## Data Availability

The data presented in this study are available on request from the corresponding author.
